# ChiVariARIBA: a modular, editable workflow and database for characterising chitin gene variation in Vibrio spp. and related bacteria

**DOI:** 10.1099/mgen.0.001439

**Published:** 2025-07-18

**Authors:** Evan P. Naughton, Matthew J. Dorman

**Affiliations:** 1 School of Mathematical and Statistical Sciences, College of Science and Engineering, University of Galway, Galway, H91 TK33, Ireland

**Keywords:** ARIBA, bacterial genomics, chitin, chitin metabolism, gene database, natural competence

## Abstract

Chitin is a highly abundant biopolymer of bioeconomic, biochemical and commercial importance. This carbohydrate is a source of nutrients for chitinolytic bacteria and can influence natural competence, surface adsorption and other fundamental aspects of prokaryote physiology. Bacterial enzymatic degradation of chitin is mediated by a well-studied set of hydrolytic enzymes, transcriptional regulators and carbohydrate transport proteins. Many of these gene products have been functionally characterized *in vitro* or *in vivo*, but there is a reliance on *in silico* genomic approaches to study the variation of these metabolic components amongst diverse bacteria. Computational surveys of bacterial genomes to date have tended to focus on determining the presence and absence of chitin metabolism genes in diverse genomes, but not on the diversity of sequences amongst these gene families. To enable future research into chitin metabolism variation in vibrios and other bacteria, we present ChiVariARIBA, a workflow for extracting chitin metabolism genes from published genome sequences of chitinolytic *Vibrio* species and their relatives, compatible with the rapid gene-finding and variant-characterizing tool ARIBA, with which to describe the presence of chitin-metabolising genes in genomes of interest and to characterize the sequence variation of these genes across diverse bacteria.

**Impact Statement**

Chitin is an economically and ecologically important biopolymer. Understanding how it is metabolized by different bacterial species – and how the complement of enzymes involved in this process varies across these species – is important for research into horizontal gene transfer through natural competence, biocontrol and the cycling of carbon and nitrogen in the circular bioeconomy. This manuscript provides a concise summary of a detailed workflow for extracting a curated list of chitin metabolism genes from published bacterial genomes and for formatting these as an easily interrogated database compatible with a commonly used rapid gene-finding tool. Alongside this project’s value to chitin metabolism researchers, we also highlight the general applicability of this workflow and its potential utility to the general microbial genomics community – we expect that this method can, in principle, be adapted to build ARIBA-compatible databases of genes involved in a wide range of other biochemical pathways or genetic regulation in bacteria.

## Data Summary

The authors confirm that all supporting data, code and protocols have been provided within the article or through supplementary data files.

**Fig. 1.**
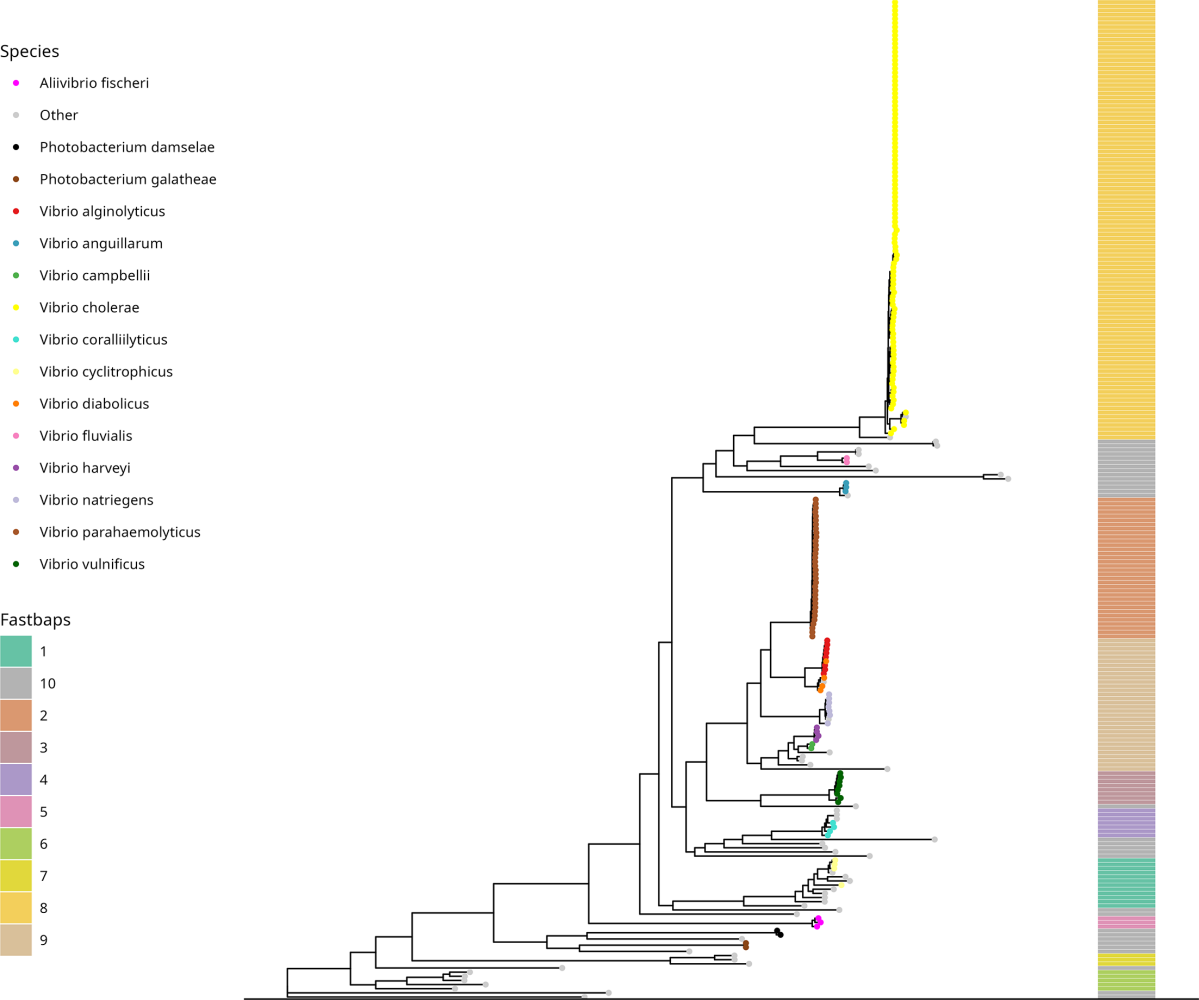
A maximum-likelihood phylogenetic tree of 241 publicly available *Vibrio* spp. genomes, computed as described in the ‘Methods’ section. The tree is rooted on ‘*Candidatus* Enterovibrio escicola’ (GCA_002464935.1 [100]). Species names as recorded in GenBank for each sample are reported (the top 15 most frequent species names are reported; all other species have been collapsed into ‘other’). FastBAPS level 1 clusters are indicated, calculated from parsimony-informative SNVs as described in the ‘Methods’ section. A horizontal black bar has been added to the bottom of this image to aid interpretation and alignment of FastBAPS cluster labels with the base of the phylogeny. This tree has been reproduced with a scale bar in, and the original tree file is available in File S4.

**Fig. 2.**
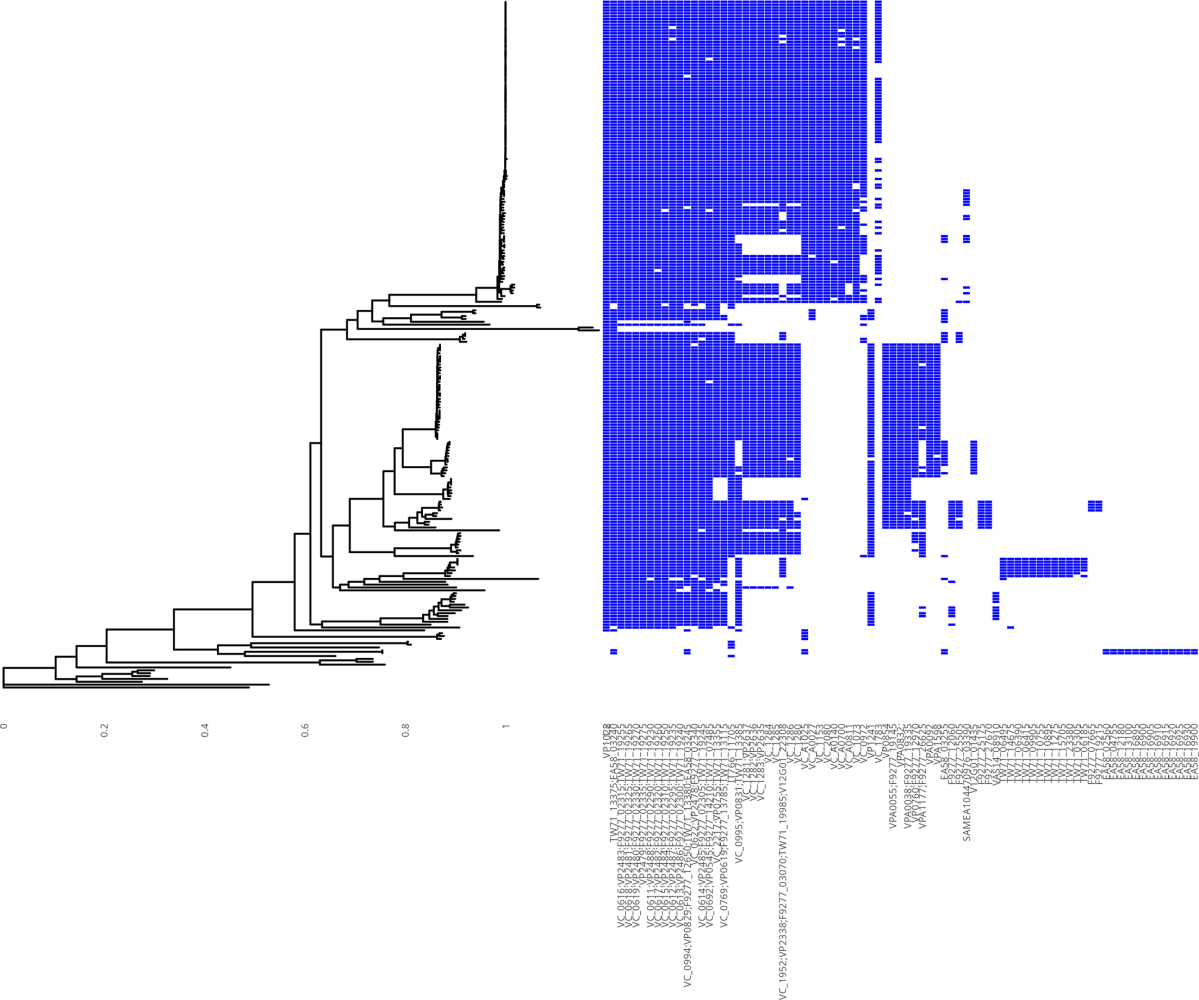
The distribution of chitin metabolism gene families across the phylogeny presented in Fig. 1. Gene family presence in a given isolate is indicated in blue; the absence of a gene family is indicated by white. Gene families are ordered by frequency from left to right; the most frequently observed families are presented to the left of the figure. Gene families are reported on the X-axis. These names can be matched to locus ID numbers using Tables 1 and S2. The code to reproduce this figure is available in File S7.

**Fig. 3.**
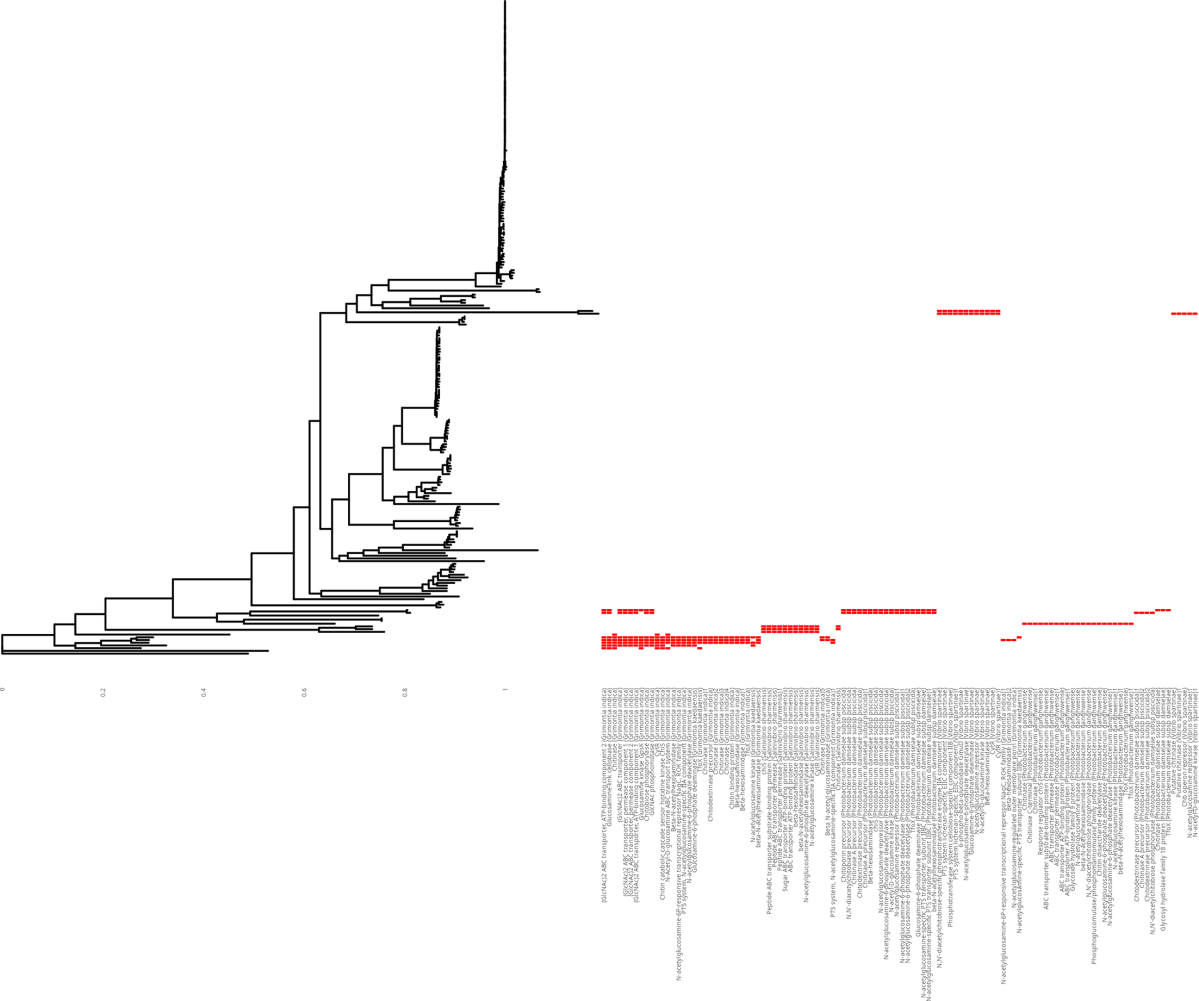
Distribution of putative chitin metabolism genes across the genus-level phylogeny presented in Fig. 1. This figure displays the presence and absence of all annotated gene families in the Panaroo pangenome linked to chitin metabolism (see the ‘Methods’ section), minus those families which contained genes known to be involved in chitin metabolism and the distribution of which was visualized in Fig. 2, in the 241 isolates used in this study. Gene families are ordered by frequency from left to right; the most frequently observed families are presented to the left of the figure. Gene family names are reported on the X-axis. The code to reproduce this figure is available in File S7.

1. No whole-genome sequencing data were generated in this study. Metadata, accession numbers and references for each genome included in this study are available in Table S1, available alongside the manuscript and deposited in Figshare: https://dx.doi.org/10.6084/m9.figshare.28343900
2. The chitin metabolism genes identified from the literature and used in the initial steps of compiling the reported database are detailed in Table S2, which is available alongside the manuscript and deposited in Figshare: https://dx.doi.org/10.6084/m9.figshare.28343900. The multiFasta file containing the sequences of these genes is available as Supplementary File S1 and has also been deposited in Figshare: https://dx.doi.org/10.6084/m9.figshare.28343900
3. A snapshot archive of the 241 annotated genome sequences used in this study has been deposited in Figshare, File S2: https://dx.doi.org/10.6084/m9.figshare.28343900
4. The ChiVariARIBA database described in this manuscript has been included in Figshare, File S3: https://dx.doi.org/10.6084/m9.figshare.28343900
5. Other intermediate files including the phylogenetic tree presented in Figs 1–3 (File S4), the parsimony-informative SNV alignment used for FastBAPS analysis (File S5), the Panaroo pangenome output files (File S10) and gene family presence/absence matrix (File S6), and other intermediate files used to produce the figures and resources reported in the manuscript have been deposited in Figshare: https://dx.doi.org/10.6084/m9.figshare.28343900
6. Multiple sequence alignments for each chitin metabolism gene family (File S8), the gene trees computed for each alignment (File S9, see Methods), and tanglegrams comparing each gene tree with the phylogeny presented in Figures 1-3 (File S11) have been deposited in Figshare: https://dx.doi.org/10.6084/m9.figshare.28343900
7. Text describing parsing scripts used in this study is available in Text S1, available alongside the manuscript. All scripts and code needed to repeat the analyses reported, and to re-generate the gene database, have been collated (File S7) and deposited in Figshare: https://dx.doi.org/10.6084/m9.figshare.28343900
8. The ChiVariARIBA database and linked code is available in GitHub: https://github.com/evannaughton/ChiVariARIBA

**Table 1.** A summary of key chitin metabolism genes from *V. cholerae* and *Vibrio parahaemolyticus*, after [1,9, 11, 30, 36]. Locus IDs given are drawn from the N16961 and RIMD2210633 annotations [38,82]. Note that this list is not exhaustive; as described elsewhere in the manuscript, genes were included in this study if their roles in chitin metabolism were supported by functional or experimental data or were commonly included in previous genomic reports

Gene with N16961 (RIMD2210633) locus ID	Description	Select references
*VC0972 (VP0760)*	Chitoporin	[33,83]
*VC1952 (VPA1177)*	Extracellular chitinase ChiA-1	[49,84]
*VCA0027 (VPA0055)*	Extracellular chitinase ChiA-2	[37,49, 85]
chiA-3 (VPA1177)*	Extracellular chitinase ChiA-3 (absent from N16961)	[35,49]
*VC0612*	*N*,*N*-Diacetylchitobiose phosphorylase	[86]
*VC0613 (VP2486)*	Periplasmic beta-*N*-acetylglucosaminidase	[87]
*VCA0700 (VP0832)*	Periplasmic chitodextrinase	[88]
*VC0614 (VP2485)*	Glucosamine-specific kinase	[89]
*VC0615*	Cellobiase, endoglucanase	[1,90]
*nagA VC0994, VC1783 (VPA0038)*	*N*-Acetylglucosamine-6-phosphate deacetylase	[36,91,93]
*nagB VCA1025 (VP0829)*	GlcN-6-P deaminase	[36,91,94]
*nagC VC0993 (VP0828)*	Represses GlcNAc metabolism genes	[92]
*nagE VC0995 (VP0831)*	*N*-Acetylglucosamine-specific transporter II^Nag^	[36,91,93]
*chiS VC0622 (VP2478)*	Governs the expression of its regulon of chitin catabolizing genes, including chitinases, a type IV pilus and a chitoporin	[1,30, 95]
*cbp VC0620 (VP2479)*	Periplasmic chitin-binding protein repressing ChiS activity	[1,95, 96]
*chb* operon *VC0620-0611 (VP2479-VP2488)*	*VC0619-0616* encodes an ABC-type transporter for (GlcNAc)_2_; products of *VC0611-0615* and *VC0620* discussed above	[1,9]
*cytR VC2677 (VP0252)*	Positive regulator of competence and chitinase expression	[50,97, 98]
*tfoX VC1153 (VP1241)*	Mediates GlcNAc_6_ utilization through CytR	[31,98]
*tfoS VC2080 (VP0854)*	Transmembrane transcriptional regulator, activates chitin-dependent gene expression	[49,51, 52]
*VC0692*	*β*-Hexosaminidase (characterized in *V. furnissi*)	[1,37, 99]
*VC2217*	Predicted exochitinase	[37]
*VC0995, VC1282*	PEP-dependent phosphotransferase transporters	[37]
*VC1073, VC0769*	Predicted chitinases	[37]

*Note that the translated product of *VPA1177* is 100% identical to the VpChi48 chitinase protein (GenBank accession number KOH17302.1) recently reported by Deng *et al.* [80]. VpChi48, VPA1177 and ChiA-3 are therefore likely to be orthologous chitinases.

GlcNAc, *N*-acetylglucosamine; GlcN-6-P, glucosamine-6-phosphate.

## Introduction

Chitin is one of the most abundant biopolymers on Earth. It has been estimated that more than 10^11^ tonnes of chitin are produced annually in the oceans [1,2], and this polymer is of commercial and economic value; the global market for chitin and its chitosan derivative was estimated to be worth USD $2.4 billion in 2016 [3,4]. Chitin consists of *N*-acetyl-d-glucosamine (NAG) monomers bonded through ß-1,4 linkages [2,5] and exists in three allomorphs: *α*-, ß- and *γ*-chitin [3,6]. Chitin catabolism into its constituent oligosaccharides provides a source of carbon, nitrogen and energy [7]. The ubiquity and global abundance of chitin also mean that its metabolism forms a key aspect of ecological carbon cycling [8,9], and given the structural rigidity and strength of this biopolymer, it follows that enzymatic degradation of chitin into constituent monosaccharides and oligosaccharides forms a part of the lifecycle of this molecule. Multiple microbial species are capable of enzymatically degrading and metabolizing chitin and its derivatives [5], including *Vibrio* spp. (it has been suggested that all *Vibrio* species are capable of metabolizing chitin [10] and that all *Vibrionaceae* are capable of growing on chitin as a substrate [11]), *Serratia* spp. (e.g. [12,13]) and *Streptomyces* spp. (believed to have acquired chitinase genes from plants [14,15]).

The interactions between bacteria and chitin (and its derivatives) have been associated with microbial pathogenesis. Chitosan has been recently shown to inhibit intestinal colonization and severity of *Vibrio cholerae* infections in a murine model [16], and a chitin-binding protein, GbpA, has been shown to mediate *V. cholerae* colonization of chitinaceous surfaces such as copepod exoskeletons [17,18]. Not only have chitinase enzymes been implicated in the pathogenesis mechanisms that cause mammals to be infected by bacteria [19], these enzymes have a range of potential applications for biocontrol [15,20, 21] including the hydrolysis of fungal cell walls and those of plant pathogens [5], the recycling of chitinaceous waste from fungiculture (e.g. reviewed in [22]) and of shellfish waste by its hydrolysis into oligomers [23,24] and energy production [25]. There are distinctions drawn between the chitin metabolism capabilities of terrestrial and aquatic origin [26]. Chitin and its metabolism play other fundamental roles in *Vibrio* ecology, including but not limited to metabolism, natural competence and biofilm formation [27,28]. The products of chitin’s enzymatic degradation regulate the natural competence phenotype in *V. cholerae* and related bacteria; competence, transformation and chitin metabolism are therefore interlinked at the level of transcriptional regulation in *V. cholerae*, in part through TfoX, the major regulator of transformation which responds to chitin-derived signals [29,31].

Consistent with the economic and biological importance of chitin metabolism, the chitin degradation pathway has been studied in detail in numerous bacterial species. The biochemistry of this degradation pathway was initially described in *Vibrio furnissi* and then in other vibrios (e.g. [32,34], reviewed in [10]). Substantial knowledge has been accumulated about this catabolic pathway, the details of which have been described in detail by others, as have diagrams describing how pathway components interact (or are believed to interact) in these bacteria (e.g. [1,9, 11, 35, 36]). It is also well-established that key enzymatic steps in the chitin metabolism process can be performed by diverse enzymes – for example, as many as eight putative endochitinases have been identified in *V. cholerae* genome sequences [1,11, 35, 37], though not all of these putative enzymes have been characterized experimentally [37]. A non-exhaustive list of the major proteins involved in *Vibrio* chitin metabolism is available in Table 1, along with key citations and a brief description of their functions (Table 1).

Previous research into chitin metabolism in *V. cholerae* has focused on a small number of reference strains, including the serogroup O1 biotype El Tor strains N16961 [1,38] and A1552 (e.g. [31]). Gene identifiers from the N16961 reference genome [38] are commonly used to identify components of this metabolic pathway in *V. cholerae* [11] (Table 1), and we have tried in this manuscript to use these identifiers for consistency and to enable cross-reference to original reports. Similar work on chitin metabolism has been done in the related species *V. parahaemolyticus*, including very recent functional work aiming to identify exhaustively the genes involved in chitin metabolism in this species [9]. *In silico* studies on the variability of ChiA/ChiA-like chitinases and other chitin metabolism genes in non-*Vibrio* marine bacteria have been performed recently, such as *Synechococcus* and *Prochlorococcus* species [39].

Whilst much is known about the ability of different bacterial species to produce chitinases and to metabolize chitin, comparatively less is known about the sequence diversity of the genes encoding the enzymatic components of these pathways – in particular, there is a paucity of information about how much these genes vary within species, as well as between species. Previous studies have used pangenome-based methods to explore the distribution of chitin metabolism genes amongst limited numbers of diverse genomes of *Vibrio* and related species [11], and the distribution of these genes within larger numbers of genomes of a single species (e.g. *V. cholerae* [35]). In that study, analysing large numbers of diverse *V. cholerae* enabled the discovery of additional chitin metabolism pathway components, but the work was limited to focusing on chitin metabolism gene presence/absence, rather than considering in detail the sequence diversity of chitin metabolizing genes in *V. cholerae* or other species and genera.

In this project, we developed a tool to address this knowledge gap, by collating a curated list of genes involved in chitin metabolism and generating a workflow with which to characterize specifically the chitin metabolism genes found in diverse *Vibrio* genome sequences. Although databases exist which couple microbial genomics to metabolic and catabolic pathways, such as BioCyc [40] and KEGG [41] (both of which contain records relating to chitin metabolism), we elected to create a specific and bespoke list of relevant genes and their functional annotation, as is commonly performed for genes involved in virulence and antimicrobial resistance (e.g. [42,44]). These smaller, focused databases are dynamic and easy to update and can contain context-specific information that is particularly relevant to users interested in chitin metabolism. Our objective was to develop a database and workflow with which to characterize gene variation within and amongst *Vibrio* spp., as well as more widely in other chitinolytic or potentially chitinolytic organisms. We chose to include only those genes for which previous experimental or functional evidence supports their having a role in chitin metabolism, and we focused on making the computational workflow easy to run and sufficiently modular to accommodate new genes as they are discovered in the future. Our aim was to enable researchers (a) to describe the *presence and absence* of chitin metabolism pathway components in genomes of interest, (b) to extract gene sequences for alignment enabling the study of how genes and their orthologues *vary* and (c) to provide curated lists of chitin metabolism-associated genes and a high-quality genome collection as baseline datasets, to enable researchers to study new genomes in terms of chitin metabolism gene presence, absence, variation and phylogenetic distribution.

We present ChiVariARIBA, a workflow to generate a simply structured database capturing chitin metabolism gene sequences, annotations and variation across *Vibrio* genomes and those of related species. We designed the workflow and database to be compatible with ARIBA [45], a tool designed for antimicrobial gene detection. ARIBA was selected because it is rapid and customizable, combining a mapping, alignment and local assembly approach with the capacity to accept custom databases of gene sequences and metadata [45]. It also accepts sequencing reads as input and thus can provide rapid summaries of chitin gene presence/absence without requiring the computational overhead of assembling and annotating a set of sequencing reads. Building this chitin gene-specific resource has also allowed us to collate relevant references and gene accession numbers in this manuscript, its supplementary materials and the database metadata. We hope that this will serve as a useful resource and reference for those in the research community with interests in bacterial chitin metabolism.

## Methods

### Genome collation

Genome sequences for all publicly available *Vibrionaceae* genomes (taxID: 641) available in GenBank and designated as being complete assemblies were downloaded on 10 September 2024 (818 genomes total). Since the purpose of this study was to compile a lasting and accurate biological resource, we used complete genomes initially so that we focused on assemblies from bacterial isolates linked to functional experimental data on chitin metabolism, in which we had confidence, and which were likely to possess reliable metadata. Genomes for which GFF annotation files were unavailable were removed from the analysis (582 assemblies remaining). To these assemblies, a set of 15 genomes was added (see Table S1 for accession numbers and sources, available in the online Supplementary Material). This second set of genomes was not designated as complete assemblies but was included to ensure that key species were represented in the dataset (see below).

We sought initially to include representatives of as many relevant *Vibrio* species as possible, but also to balance this desire against the need to respect the possibility that other groups might have deposited genomes in GenBank prior to reporting their own peer-reviewed publications. We removed any genomes which could not be associated with a published manuscript in PubMed from our analysis. However, five genomes that were publicly available in GenBank represented key species of importance to wider taxonomic diversity, but we were unable to associate them with a peer-reviewed publication (Table S1). To ensure the representation of these species of potential interest, we exceptionally retained these five genomes for subsequent steps in database generation, but we note that each of these genomes was publicly available in GenBank at the time of dataset compilation, and we have not sought to draw any further conclusions about these specific genomes.

Additional quality control steps were performed to identify and remove genomes which were unannotated or of poor quality – flagged by NCBI as containing ‘many frameshifted proteins’ or having failed a completeness check, and which were later found to prevent pangenome construction – and were removed. The latter were removed using two custom scripts (frameshifts_count.py and name_and_remove_frameshift_files.py; File S7, see Text S1 for details of these scripts). Annotated genomes were converted to Prokka annotation format using a modified version of convert_refseq_to_prokka_gff.py (https://github.com/gtonkinhill/panaroo/blob/master/scripts/convert_refseq_to_prokka_gff.py; the modified script is available in File S7) for compatibility with the pangenome calculation software Panaroo [46]. These genomes were pre-processed using panaroo-QC, and after manually reviewing the contig number, gene count number and genome similarity estimates for each assembly, a total of 241 high-quality genomes remained for use to compute a pangenome. The species distribution of these genomes was biassed towards *V. cholerae* (104), *V. parahaemolyticus* (34) and *Vibrio vulnificus* (8), consistent with the distribution of *Vibrio* species genomes in GenBank.

### Initial collation of representative chitin metabolism gene sequences

From the published literature, a list of genes involved in chitin metabolism in *V. parahaemolyticus*, *V. cholerae*, *Vibrio coralliilyticus*, *Photobacterium galatheae*, *Vibrio harveyi* and other members of the *Vibrionaceae* was compiled (Table 1; e.g. [9,11, 35, 37, 47,49]). Representative sequences for a total of 179 genes were extracted manually from annotated genome assemblies, guided both by gene accession numbers/locus tags (Table 1) and annotations containing keywords such as ‘chitin’. These were used to create a multiFasta-formatted list of gene sequences (published_chitin_genes.fasta; File S1). The multiFasta file headers contain the accession number of the source genome and the chromosomal co-ordinates of the extracted gene. This initial list of genes was limited to those which were known to be involved in chitin metabolism, having been experimentally or functionally validated, or otherwise implicated in chitin metabolism in a way that did not rely solely on automatic genome annotation or function prediction (see Tables 1 and S2 for gene accession numbers and further details). As well as metabolism genes, the *tfoX*, *tfoS* and *cytR* genes, which encode master regulators of the natural competence pathway and machinery in *V. cholerae* and are therefore linked to chitin metabolism [31,50,53], were also included to ensure that the regulatory components of chitin metabolism were reflected in the database. Similarly, select genes implicated in chitin metabolism in non-*Vibrio* marine bacteria, such as *nagK* [39], were included. Upon manual inspection, the gene EA58_07035 from * P. galatheae* S2753 [48], annotated as coding for a chitodextrinase, was determined to be a pseudogene and was excluded from our analysis. After these edits, the published_chitin_genes.fasta file contained a total of 189 genes (File S1).

### Pangenome calculation and extraction of chitin gene diversity from gene families

In order to capture the diversity of chitin metabolism genes across the 241 diverse representative genomes, a pangenome was constructed from which gene families containing the sequence diversity of chitin metabolism genes could be extracted. A pangenome was calculated using annotated genomes and Panaroo v1.5.1 [46], using options '--clean-mode strict --alignment core --aligner mafft --core_threshold 0.95 --remove-invalid_genes -f 0.6'. The default protein identity threshold for including genes in a gene family is 0.7; initial inspections found that this classification was too stringent, over-partitioning genes into multiple gene families. An example of this was the gene family to which *VC1282*/*VP2636* belongs. This gene (also known as *celB*) encodes an experimentally proven component of the (GlcN)_2_ cellobiose PTS transporter [30]. At a threshold of 0.7, the gene family was reported as absent in isolates which were known to be capable of metabolizing chitin. Manual inspection showed that the gene family had been partitioned into two families rather than one. Reducing the threshold to 0.6 rectified this over-partitioning and created a single gene family; thus, the threshold selected for downstream analysis was '-f 0.6'. Pangenome analysis results are available in File S10.

MakeBLASTDB [54] was used to generate a blastn database from the Panaroo output file combined_DNA_CDS.fasta (-dbtype nucl). This output file contains all of the nucleotide sequences for annotated genes, as well as those genes re-found by Panaroo [55]. The file of published chitin metabolism gene sequences was used as a blastn query against this database (-outfmt 6; default BLASTn length and identity cut-offs). A derivative of the gene sequence file in which the fasta headers had been post-processed using truncate_fasta.py (File S7) was also used to query the blast database to determine subject sequence mismatches relative to the queries (-outfmt ‘6 qseqid sseqid btop’ -dust no -parse_deflines). A custom script (convert_btop_to_ariba_compatible.py, File S7) converted the BTOP strings into a format compatible with ARIBA. Data for each of the gene families which were identified by this blast search were extracted from the Panaroo gene_data.csv output file and merged with the blast results (merge_blast_files.py, File S7). Since similar chitin metabolism genes had been included from multiple species, redundant or duplicate genes were excluded, producing a final file containing (a) each chitin metabolism gene’s nucleotide and translated protein sequence, (b) annotation information and (c) accession number and other information relating to the source genome from which the gene was extracted. This combination of information from single genes and the requisite metadata with which to link those genes to the wider gene diversity in the pangenome formed the basis of the ARIBA database built subsequently. Post-processing steps were performed to merge the files and produce a nucleotide sequence and metadata table compatible with ARIBA v2.14.6 [45]. Variants in chitin metabolism genes that were detected by ARIBA relative to the reference database were summarized into a bespoke report using a custom script (collapse_report.py; File S7). ARIBA gene detection thresholds can be customized by the user at the ‘ariba run’ step; throughout the work reported in this manuscript, default ARIBA parameters were used for alignment identity (90%), alignment length (20%) and assembly coverage (50×).

### Population structure assessment and clustering

A maximum-likelihood phylogeny of the 241 samples included in this dataset was generated using the core-gene alignment produced by Panaroo of 143 core genes and 68,014 sites, of which two sites were constant, 63,161 were parsimony-informative and 4,851 were singletons. Details of the genes included in the alignment are available in the core_alignment_header.embl file, part of File S10. The number of invariant sites in the alignment and their proportional allocation to A, T, G and C was determined using SNP-sites v2.5.1 [56] ('-C core_gene_alignment_filtered.aln'). An SNP-only alignment was produced from this core-gene alignment using SNP-sites v2.5.1 [56] and used alongside the invariant site proportion data from the core-gene alignment as input to generate a phylogeny using IQ-Tree2 [57] ('-fconst 16066,11423,17035,15462 -m MFP -nt AUTO -B 1000'). ModelFinder [58] was used to select the best-fit evolutionary model for the input data (TIM2+F+R10).

FastBAPS v1.0.8 [59] was used to cluster genomes agnostic of their phylogenetic position. An alignment of 44,612 parsimony-informative SNPs was extracted from the SNP-only alignment using extract_PI_SNPs.py (https://gist.github.com/jasonsahl/9306cd014b63cae12154) and used as an input alignment for FastBAPS under an optimized symmetric prior (optimized hyperparameter: 0.029; code available in File S7). Concordance between the phylogeny topology, species assignment based on sample metadata and FastBAPS clusters was assessed by visualization (Fig. 1).

### Gene trees

Gene trees were calculated using IQ-TREE2 and gene family multiple sequence alignments as input, using ModelFinder to select the most appropriate phylogenetic model for the input data. Along with a tanglegram comparing the gene tree with the species tree (collated in File S11, simultaneously providing information on gene family presence/absence within the wider population as well as the variation within that family represented in the gene tree), the gene family alignment and phylogenetic model used to compute each tree are recorded in the IQ-Tree output file and have been provided (File S9).

### Additional information

Additional R packages used in this study, which are required for the custom scripts published alongside the manuscript, were SeqInr v4.2-36 [60], ape v5.8 [61], ggplot2 v3.5.1 [62], ggtree v3.12.0 [63,64], stringr v1.5.1 [65], ggnewscale v0.5.0 [66], tidyr v1.3.1 [67], dplyr v1.1.4, tidyverse v2.0.0 [68], gridExtra v2.3 [69], reshape2 v1.4.4 [70], pheatmap v1.0.12, treeio v0.4.6 [71], RcolorBrewer v1.1-3 [72], tibble v3.2.1 [73] and phytools v2.30 [74].

## Analysis results and outputs

### ChiVariARIBA produces bespoke ARIBA reports containing chitin metabolism-specific information

Using this workflow, the ARIBA database and a set of sequencing reads will generate a report in CSV format, containing chitin metabolism-specific information about detected genes and their predicted products. These domain-specific data include reference genome protein locus IDs where known (see also Table 1), facilitating persistent cross-referencing to existing literature. The gene family names from the pangenome used to build the database are included in the report, enabling a researcher to extract easily any relevant additional information from the files contained in this manuscript and its repositories. Functional annotation information for gene products has also been extracted from the annotated genomes used to build the pangenome. The accession numbers and chromosomal co-ordinates of the reference genes used to build the database can also be recovered (File S1, Table S2), and custom scripts have been included alongside this manuscript which will produce a collapsed list of the sequence variants in a chitin metabolism gene from a new genome of interest relative to these reference gene sequences (see the ‘Methods’ section, File S7). Other gene-specific statistics are also reported, including the length of queries and results, and the position and number of sequence variants.

### Core chitin metabolism genes are conserved amongst *Vibrio* and related species

A pangenome built using the 241 genome sequences described above yielded a total of 113,832 gene families. The population structure of the 241 genomes included in the analysis was assessed by computing a core gene phylogeny from an alignment of 143 core genes (see the ‘Methods’ section). The number of genes in the core-gene alignment is small; this is because the dataset contains several diverse species, and our aim was to select pangenome parameters which balanced core genome definition against the need to create meaningful gene families for detecting chitin metabolism genes of interest. Therefore, in addition to a phylogenetic approach, we also assessed population structure using the FastBAPS tool [59] which assigns samples to clusters without reliance on the phylogenetic tree. Level 1 BAPS clusters were found broadly to correspond to the taxonomic assignments (Fig. 1), and the phylogeny and BAPS clusters were therefore determined to have recapitulated the clustering of *Vibrio* and *Vibrio*-related bacteria into species, as defined by the taxon IDs assigned to each sample in GenBank (Fig. 1, Table S1).

The presence and absence of chitin metabolism-associated gene families were determined by identifying the pangenome gene family into which each of the 189 representative genes (File S1) had been placed and mapping the distribution of the gene family across the core-gene phylogeny for each gene family (Fig. 2). Consistent with previous reports [11], certain chitin metabolism genes are core to the vast majority of *Vibrio* [e.g. *VP1028* and *VC0614-0619*, coding for (GlcNAc)_2_ transport proteins [11]; Fig. 2]. Other genes display a species-specific distribution, such as those present nearly exclusively in *V. cholerae* and absent from other species (*VC2080*, *VCA0140*, *VCA0700*, *VCA0811*, *VC1073* and *VC0972*; Fig. 2).

We initially thought that this observation was inconsistent with previous reports – Hunt *et al*. had reported that *VC1073* and *VCA0700* are both copies of a chitodextrinase gene, present in at least one copy in all of the *Vibrio* spp. included in their study [11]. *VC1073* and *VCA0700* each encode one of the seven *V. cholerae* chitinase genes studied by Hayes *et al*. [37] (Table 1). We therefore had anticipated that these genes would be distributed across more species than *V. cholerae* alone. However, we found that *VPA0832*, encoding a functional orthologue of the *VCA0700* product in *V. parahaemolyticus* (80% blastp identity to the *VCA0700* product; accession numbers BAC62175.1 and AAF96599.1, respectively), is placed into a separate gene family to that of *VCA0700* by ChiVariARIBA, and the distribution of the gene families containing *VCA0700* and *VPA0832* is mutually exclusive; thus, the *V. parahaemolyticus* gene family is not detected in *V. cholerae*, and vice versa (Fig. 2). This indicated strongly that our method and approach were correctly classifying genes into families that showed both ubiquitous and species-specific distribution.

### The ChiVariARIBA workflow captures the diversity within genes, as well as gene presence/absence across a collection of genomes

A particular advantage of using this workflow is its ability not only to detect the presence and absence of a curated set of chitin metabolism genes in an isolate of interest but also to provide information about the sequence diversity of gene families across the diversity of *Vibrio* spp. The pangenome output and the list of genes involved in chitin metabolism collated from the literature (see the ‘Methods’ section) were used to extract multiple sequence alignments for each gene family containing a gene represented in the chitin metabolism gene list. These alignments can be used to study the diversity of each gene family at the nucleotide or translated protein level, and the reports generated by the workflow will include information about how a gene found in a sample of interest varies relative to the reference gene in its family, as well as the wider gene family. Phylogenetic trees can be computed using the gene family sequence alignment, to which gene variants can be added after detection using ChiVariARIBA. Tanglegrams can also be generated to compare simultaneously the distribution of a gene of interest across the phylogeny used in Figs1  2, and the variation in a gene family to that of the wider phylogeny (Files S8 and S11).

### ChiVariARIBA has scope to identify genes additional to those included in its database

Several isolates included in the analysis appeared to lack chitin metabolism genes (Fig. 2). This, in principle, is inconsistent with the previously mentioned position that all vibrios are believed to be able to metabolize chitin [10] or grow on chitinaceous substrates [11]. We speculated that rather than lacking the chitin metabolism pathway, these isolates – which are more distantly related to the well-studied *V. cholerae* and *V. parahaemolyticus* species (Fig. 2) – might instead possess gene orthologues which were not included in the gene families with known chitin metabolism genes because of the similarity thresholds used in our analysis. The fact that comparatively few genes were designated as ‘core’ in the pangenome analysis strengthened this suspicion.

To confirm this, we inspected the annotated assemblies which appeared to lack chitin metabolism genes (Fig. 2) and found that these genomes were indeed predicted to harbour genes annotated as being involved in chitin metabolism, including putative chitinases, *cytR* and *tfoX* regulators and *nag* operon components. We identified the gene families into which these genes had been placed in the pangenome output and created a new figure highlighting the distribution of these families across the genomes under consideration (Fig. 3). We ensured that these families did not include the families already included in Fig. 2 [i.e. genes known to be involved in chitin metabolism (Table S2)], finding that isolates in which no ‘known’ chitin metabolism genes were found did harbour ‘putative’ chitin metabolism genes (Fig. 3). It was striking that this appeared to be mutually exclusive (Figs2  3) – isolates which lacked ‘known’ genes possessed complements of ‘putative’ genes, and vice versa. This observation is consistent with the expectation that *Vibrio* spp. should be chitinolytic [10,11] and indicates that there is an undescribed diversity of chitin metabolism genes across diverse species. This is further reinforced by our testing the workflow using sequencing reads from *Synechococcus* and *Prochlorococcus* species [39]. These species are chitinolytic, but no reads were determined to map to the reference database, indicating that although there is a functional overlap between the ability of these species to metabolize chitin with the species used to build the database, this is unlikely to be due to the sharing of genes with similar sequences between these diverse species. We also observed that the genome assembly for *Vibrio gazogenes* (GCA_002196515.1 [75,76]) lacked chitin metabolism-associated genes, except for *nag* operon gene family members.

Further exploration of this diversity is beyond the scope of this work, which focused on constructing a tool and database relying on functionally validated chitin metabolism genes. As such, the database is likely not to capture the totality of chitin metabolism genes from the genomes under study, but we feel that the decision to exclude genes about which there is uncertainty or ambiguity around their function – and to highlight this fact in the manuscript – will give researchers increased confidence in the results obtained from using the database. This implies that automated annotation may have the capacity to identify genes that are not yet functionally confirmed to have roles in chitin metabolism, or which have diverged so far at a nucleotide level from the genes in the database that Panaroo did not place them into the same gene families.

## Future applications

The recent link between chitosan and *V. cholerae* virulence [16], together with the well-established role for chitin in *V. cholerae* natural competence [1,29, 31, 52] and biofilm formation [77,79], points to these polymers and their derivatives as being of fundamental importance to *V. cholerae* biology and – potentially – to the biology of other vibrios.

With these research areas in mind, we have developed a focused database, using genes from the literature which have been implicated functionally in the chitin metabolism pathway, to enable future research into the biology of vibrios and of other bacteria, without reliance on automated prokaryotic annotation pipelines alone. We have deliberately elected only to include genes in the database which are known to be involved in chitin metabolism, rather than to include putative genes or those predicted to be involved in the metabolic pathway from sequence alone. Not only does the database contain reference sequences from the literature for functionally validated chitin metabolism genes, but it also contains all high-confidence variants and alleles of each chitin metabolism gene found in the genomes studied. We also anticipate that the database will be easily expanded in the future to accommodate new genes, perhaps as suggested by the community, or perhaps in response to new publications. Recent research in the area of bacterial chitin metabolism [9] and in characterizing novel chitinases in *Vibrio* [80] indicates that this is an area of current focus, and we welcome suggestions from the community for future revisions to the database.

We also anticipate value in using this method in other chitinolytic bacterial species which are not members of *Vibrionaceae*. This will require a similarly meticulous approach to be taken to the quality control of composite genome sequences, as well as the choice of analysis tool thresholds; it cannot be assumed that the cut-off values used for these *Vibrionaceae* will necessarily be applicable to all chitinolytic organisms (e.g. terrestrial chitinolytic bacteria). Other benefits of ChiVariARIBA to users include its potential utility for scanning metagenomic sequencing reads for chitin metabolism genes, as well as the speed of its use with ARIBA (5–6 min on a single CPU thread during testing; Dell Latitude 5450 Intel Core Ultra 5 125U, 16 GB RAM) compared to needing to complete a SPAdes v4.2.0 *de novo* assembly (~22 min on the same machine) prior to scanning assemblies for chitin metabolism genes using blast-based approaches (e.g. using ABRicate [81] or similar). Although we have focused on making the gene database compatible with ARIBA at this time, we also anticipate that it can be easily adapted to tools which screen assembled genomes at the contig level (e.g. ABRicate [81]) in future revisions or by other researchers.

The relative ease with which new genes can be included in this database makes it tuneable and customizable for other researchers. The results presented in Fig. 3 also speak to the importance of considering the inclusion of genes that have not yet been characterized functionally in future iterations or revisions of ChiVariARIBA. We have chosen not to include these genes at present, chiefly to increase the likelihood that genes detected are in fact truly involved in the biochemical process, but we also anticipate that as the community makes use of and alters this database, a user may well find it useful to include these genes for their purposes. In the spirit of rendering the database and its underlying data findable, accessible, interoperable and reusable, and of maximizing the utility of the tool to other researchers, we have tailored the workflow’s reports and outputs for usability, and all of the scripts needed for the processing of intermediate data files have been released alongside the database. This maximizes the sustainability and opportunities for reuse of this work, both by chitin researchers and by the wider research community.

## Supplementary material

10.1099/mgen.0.001439 Table S1.

10.1099/mgen.0.001439 Table S2.

10.1099/mgen.0.001439 Uncited Supplementary Material 1.

## Abbreviations

NAG: *N*-acetyl-d-glucosamine
