# Supplementary material for: ChiVariARIBA: a modular, editable workflow and database for characterising chitin gene variation in Vibrio spp. and related bacteria
Source: Microb Genom. 2025 Jul 18;11(7):001439. doi: 10.1099/mgen.0.001439 (PMC12282291; doi:10.1099/mgen.0.001439)
Supplement: Uncited Supplementary Material 1. [file mgen-11-01439-s003.pdf]

Supplementary Material for

ChiVariARIBA: A modular, editable workflow and database for characterising chitin gene variation in *Vibrio* spp. and related bacteria

Evan P. Naughton, Matthew J. Dorman

These supplementary materials include

Text S1 – Notes on the operation of Python scripts frameshifts_count.py and name_and_remove_frameshift_files.py (this file)

Figure S1 – *Vibrionaceae* phylogenetic tree including scale bar (this file)

Table S1 – metadata for genomes included in this study (.xlsx)

Table S2 – metadata for chitin metabolism genes included in this study (.xlsx)

File S1 – representative published chitin metabolism gene sequences (.fasta)

File S2 – archive of annotated genome sequences used in this analysis (.tar.gz)

File S3 – ChiVariARIBA database files (.zip)

File S4 – Newick phylogenetic tree file used to produce Figures 1-3, Figure S1 (.tre)

File S5 – alignment of parsimony-informative SNVs for FastBAPS analysis (.fasta)

File S6 – Panaroo gene presence/absence matrix (.Rtab)

File S7 – scripts used in this project (.zip archive of python, bash, and R code)

File S8 – chitin metabolism gene family multiple sequence alignments (.fasta)

File S9 – gene family trees, IQ-Tree outputs (.zip archive, various file types)

File S10 – Panaroo pangenome output (.zip archive, various file types)

File S11 – images of gene tree tanglegrams (.zip archive of .png files)

Tables S1 and S2 are included alongside this manuscript.

Files S1-S11 are stored in the Figshare repository linked to this manuscript:
https://dx.doi.org/10.6084/m9.figshare.28343900

The ChiVariARIBA database is also accessible on GitHub:
https://github.com/evannaughton/ChiVariARIBA

Text S1 – Notes on the operation of Python scripts frameshifts_count.py and name_and_remove_frameshift_files.py

During initial testing of the workflow described in the manuscript, we found that a sub-set of genomes downloaded from GenBank, when included in the input dataset for pangenome construction with Panaroo, caused Panaroo to fail. This was found to be due to the over-representation of frameshifted gene features in the annotation saved alongside the assembly in GenBank, on which our method relied.

We found that these genomes could be identified by as having a large (>100s) number of loci annotated with the specific annotation feature 'pseudogene' (see manual: https://www.ncbi.nlm.nih.gov/genbank/genomes_gff/, accessed May 2025). For example, assembly accession number GCA_023612615.1 (ASM2361261v1 – *Vibrio splendidus* – accessed May 2025) is designated as being a complete assembly, however its NCBI record highlights that the assembly "annotation fails completeness check" and has "many frameshifted proteins" (https://www.ncbi.nlm.nih.gov/datasets/genome/GCA_023612615.1/). This genome contains 1,286 genes for which the annotation contains the comment "Note=frameshifted".

Since our initial attempts to construct a pangenome using Panaroo failed due to the presence of annotated assemblies which contained large numbers of these frameshifted proteins (other examples include GCA_023612615.1, GCA_000196495.1, GCA_003544875.1, and GCA_004116385.1), we excluded such genomes from our analysis. To detect and identify these genomes, two scripts were run; frameshifts_count.py counted the number of frameshifted genes within each genome by searching for the keyword "frameshift" within each annotation. Another, called name_and_remove_frameshift_files.py, determined if more than 5 frameshifts were found per genome by searching the keyword "frameshift" and then removed assemblies meeting this criterion from the dataset.

Supplementary Figure

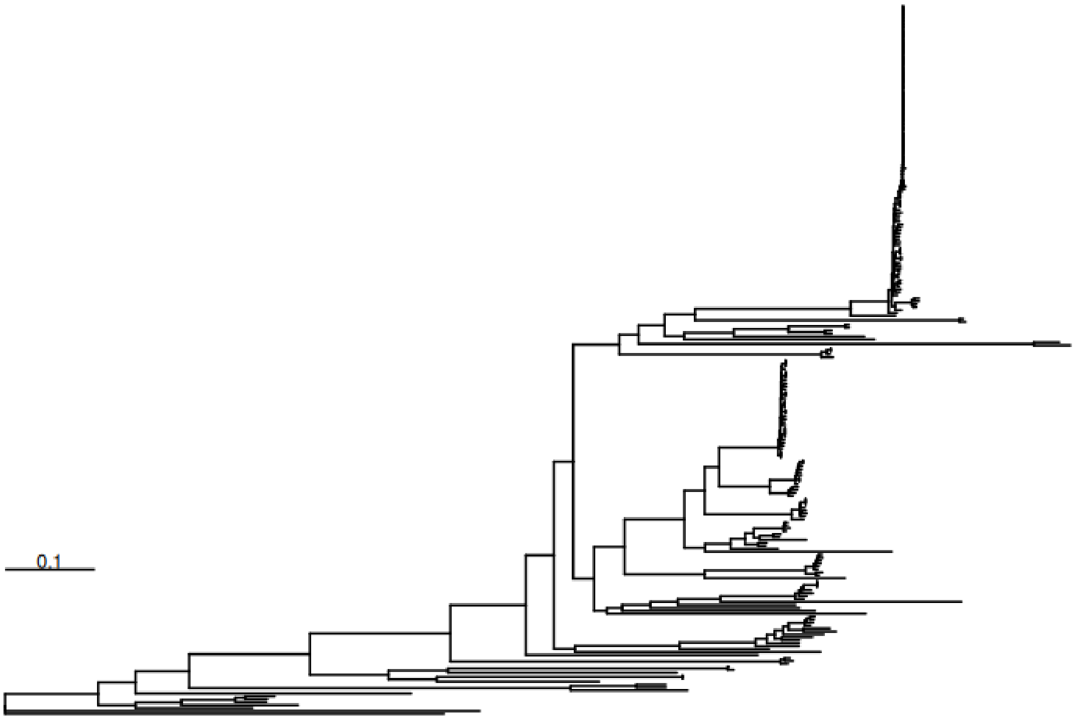

Figure S1 – *Vibrionaceae* phylogenetic tree including scale bar. Phylogenetic tree presented in Figures 2-3, including a scale bar representing the number of variations per variable site in the alignment (see Methods). The original tree file is available as Supplementary File S4.
